# Rare adipose disorders: a review of clinical presentation, pathophysiology, and management with illustrative case series

**DOI:** 10.1016/j.jpra.2026.10.014

**Published:** 2026-10-21

**Authors:** Hao Huang, Dylan K. Kim, Kathleen Gu, Terrence C. Green, Ryan Engdahl

**Affiliations:** a Department of Surgery, Division of Plastic and Reconstructive Surgery, New York Presbyterian Hospital, Cornell and Columbia University, New York, NY, USA; b Department of Surgery, Division of Plastic and Reconstructive Surgery, Harlem Hospital, Columbia University, New York, NY, USA

**Keywords:** Rare adipose disorders, Lipedema, Lipomatosis

## Abstract

Rare adipose disorders are abnormal subcutaneous adipose tissue diseases that present as a spectrum of soft tissue abnormalities that may be encountered by plastic surgeons. Presentation ranges from distinct subcutaneous masses in characteristic distributions in the body to large generalized fatty deposits. It is imperative to recognize these rare adipose disorders, as they are typically refractory to standard treatment approaches to obesity, such as weight loss, lifestyle modifications, pharmacologic therapy, and bariatric surgery. In this review, we provide a complete and updated review of lipedema, Madelung disease, familial multiple lipomatosis, and Dercum’s disease as well as present a case series highlighting our surgical approach to rare adipose disorders.

## Introduction

Obesity is characterized by an excess of subcutaneous adipose tissue, which can be effectively managed by lifestyle changes and bariatric surgery. After massive weight loss, the loose skin and subcutaneous fat also become the target of surgical body contouring procedures. In contrast, rare adipose disorders (RAD) comprise a heterogeneous group of conditions characterized by abnormal subcutaneous adipose tissue but differing in pathophysiology, clinical presentation, and management. These disorders may be broadly divided into lipedema and non-lipedema disorders, which include Madelung disease, familial multiple lipomatosis, and Dercum’s disease. RADs are distinct from obesity and do not respond to caloric limitation or increased energy expenditure. They arise from fundamental changes in physiology, such as lymphatic dysfunction in lipedema and secondary lymphatic dysfunction due to the abnormal growth of brown stem cells in Madelung disease.^1^ RADs can present as prominent and distinct adipose accumulations or subtle excess in subcutaneous fat.

Often misdiagnosed as obesity, RADs are underrecognized and undertreated due to low awareness of the disease entity. As they are usually refractory to traditional weight loss methods like diet, exercise, and bariatric surgery, they require a nuanced multidisciplinary approach that includes lymphatic decongestive therapy, medications, and often surgery.^1^ In this review, we review the current literature in the pathophysiology, clinical presentation, and treatment of lipedema, Madelung disease, familial multiple lipomatosis, and Dercum’s disease. In addition, we present a case series highlighting our surgical approach to RADs.

## Methods

Following the PRISMA guidelines, a literature review of lipedema, Madelung disease, familial multiple lipomatosis, and Dercum’s disease was conducted on the PubMed engine by the National Institutes of Health. The following search terms were used to identify an initial cohort of articles: “(Madelung) OR (benign symmetric lipomatosis) OR (multiple symmetric lipomatosis) OR (Launois-Bensaude) OR (lipedema) OR (lipalgia) OR (familial multiple lipomatosis) OR (hereditary multiple lipomatosis) OR (Dercum) OR (adiposis dolorosa).” Articles from database conception to July 2026 were included for full-text review if they discussed the epidemiology, pathophysiology, risk factors, presentation, diagnosis, and/or treatment of the four adipose disorders and were written in the English language. Reference lists of included studies and relevant review articles were also manually screened to identify additional eligible publications not captured by the database search. Given the rarity of the disorders, all types of articles, including case reports and series, cohort studies, and reviews, were included.

## Results

### Lipedema

#### Clinical presentation and diagnosis

Lipedema, or lipalgia syndrome, is a chronic, progressive disorder of subcutaneous adipose tissue characterized by bilateral, symmetric accumulation of painful fat in the lower limbs and hips, sparing the feet and hands. The tissue is tender and bruises easily and, unlike the fat of simple obesity, resists caloric restriction, exercise, and bariatric surgery.^2^, ^3^, ^4^

It occurs almost exclusively in women and typically emerges at puberty, pregnancy, and menopause.^5^^,^^6^ Its true prevalence is largely unknown due to under-recognition and misdiagnosis, which is reflected in the variability in prevalence estimates found in literature. A small occupational-screening study (n = 62) reported prevalences from 4.8% to 39% within the same cohort depending on the diagnostic threshold applied.^7^ Meanwhile, a self-selected online survey of 253 women reported a rate of 12.3%.^8^ Higher estimates may be inflated by selection bias and the lack of confirmatory clinical examinations. Furthermore, the overall heterogeneity in the data stems primarily from the absence of a validated diagnostic standard. Consequently, the commonly cited figure of 11% should not be viewed as a population-based measurement.^2^^,^^6^^,^^9^

Lipedema is a clinical diagnosis as no biomarker, imaging finding, or histologic feature is diagnostic. Ancillary testing serves primarily to exclude alternative conditions, and standardized diagnostic criteria remain lacking.^3^, ^4^^,^^10^, ^11^, ^12^ Supportive features include disproportionate symmetric fat distribution, tenderness, easy bruising, a negative Stemmer sign in early disease, and minimal pitting.^4^^,^^10^ Per 2024 S2k guidelines, a disproportionate increase in limb fat without accompanying symptoms should not be diagnosed as lipedema.^10^

Adjunctive investigations, including ultrasound-derived dermal and subcutaneous thickness cut-offs, MRI, DEXA, bioimpedance, and lymphoscintigraphy, may support the assessment but cannot establish the diagnosis.^13^ Lipedema is conventionally categorized by anatomic region (type I, hips and buttocks; type II, extending to the knees; type III, to the ankles; type IV, with arm involvement; type V, calf-limited pattern) and by morphologic stage (1 to 3, reflecting progressive skin and subcutaneous surface changes).^14^ The 2024 S2k guideline cautions that morphologic stage should not be used to gauge disease severity or to guide treatment, as symptom burden correlates poorly with surface appearance.^10^

#### Pathophysiology

The pathophysiology of lipedema is incompletely understood. Lipedema is strongly associated with overweight status and obesity, adding to diagnostic difficulty and disease burden.^15^ Familial clustering is consistent with an autosomal-dominant inheritance pattern with sex limitation and incomplete penetrance, and genome-wide association studies have implicated loci including VEGFA, GRB14-COBLL1, and LHFPL6.^5^^,^^16^^,^^17^ Histological studies reveal adipocyte hypertrophy alongside dilated dermal blood, lymphatic microvessels, and macrophage infiltration, which become more pronounced with disease stage.^18^^,^^19^ Although an increase in adipogenesis is frequently proposed, its actual contribution remains uncertain. *In vitro*, patient-derived stem cells exhibit reduced adipogenic differentiation despite *in vivo* expansion of tissue, leaving the relationship between progenitor-cell behavior and tissue growth unresolved.^19^^,^^20^

Vascular and lymphatic mechanisms are closely linked to the cardinal signs but warrant careful review. Microangiopathy with increased capillary fragility and permeability may contribute to bruising and interstitial edema.^11^^,^^19^^,^^20^ Near-infrared imaging in early disease shows dilated lymphatic vessels with preserved large collecting lymphatic function, in contrast to primary lymphedema.^21^ Conversely, lymphoscintigraphy shows delayed peripheral lymph transport in most lipedema legs.^22^ These observations can be reconciled by stage and anatomical level: early disease with micro-lymphatic dilation and preserved macro-lymphatic function, progressing to secondary lymphatic overload and lipo-lymphedema. Chronic low-grade inflammation and progressive interstitial fibrosis are well documented, accompanied by a shift toward an M2-like macrophage phenotype distinct from that observed in obesity.^18^ Further, the disease’s female predominance and time of onset at periods of hormonal change implicate estrogen and estrogen-mediated mechanisms. However, the specific mechanisms remain uncertain.^5^^,^^23^^,^^24^ Lipedema’s pain out of proportion to exam may also suggest a neurogenic component, supported by dermal nerve abnormalities and increased neurogenic inflammatory mediators.^24^

#### Non-surgical management

Conservative therapy is the first-line treatment and remains a lifelong maintenance backbone, often acting as the cornerstone of a multidisciplinary approach to treating lipedema. It combines complex decongestive therapy, comprising manual lymphatic drainage, compression, exercise, and skin care, sometimes supplemented by intermittent pneumatic compression.^4^^,^^6^^,^^20^ Although conservative management alleviates pain, reduces edema, and enhances quality of life, it cannot eradicate the diseased adipose tissue.^6^

Evidence supporting conservative treatment is limited. A systematic review found that physical activity, particularly aquatic exercise, improves symptoms and cardiometabolic health.^25^ Dietary strategies typically target inflammation and concurrent obesity rather than lipedema fat, which is resistant to caloric restriction. Evidence for anti-inflammatory and ketogenic diets is limited to case reports and pilot investigations.^2^^,^^26^, ^27^, ^28^ No pharmacologic therapy is approved or established as disease-modifying. Given the substantial burden of pain and psychological distress, multidisciplinary and psychological support are standard, and bariatric surgery reduces weight and comorbidity but has a limited and variable effect on the lipedema fat.^3^^,^^6^^,^^29^

#### Surgical management

When conservative therapy fails to control symptoms, liposuction is the definitive treatment and the only modality shown to reduce the pathologic adipose tissue, with lipectomy reserved for the most advanced disease or for accompanying skin excess.^6^^,^^14^ Lymphatic-sparing techniques, including water-assisted (WAL), tumescent (TTL/TAL), power-assisted (PAL), and ultrasound-assisted (UAL) liposuction, aim to limit iatrogenic lymphatic injury, a rationale supported by lymphoscintigraphic evidence that tumescent liposuction does not diminish lymphatic function.^22^ A 2024 meta-analysis of 906 patients (2542 procedures) found low overall complication rates (1.5% for tumescent, 4.0% for power-assisted, 0% for water-assisted, and 2.3% for combined power- and water-assisted liposuction), although heterogeneity across studies was high.^30^ Patients with lipedema who have undergone bariatric surgery may present with refractory excess adipose tissue and skin redundancy, for whom a staged approach of liposuction followed by skin excision may be required.^29^

Reported complications include contour irregularity, hyperpigmentation, seroma, hematoma, thromboembolism, and infection.^30^ A small potential risk for mortality associated with liposuction should be acknowledged as well. A recent investigation of 246,119 liposuction cases at accredited U.S. ambulatory surgery centers between 2019 and 2021 identified a mortality rate of 0.009%.^31^ Importantly, no lipedema-specific mortality rate has been published, and the largest lipedema series to date reported no deaths.^30^ However, because liposuction used to treat lipedema is frequently large-volume and staged over multiple sessions in patients who often have obesity and other comorbidities, the extrapolated perioperative risk is non-negligible and warrants explicit acknowledgment and mitigation. Risk reduction includes careful patient selection, perioperative optimization, potential thromboprophylaxis, and input from lymphology, physiotherapy, nutrition, and psychology.^3^^,^^6^^,^^11^

Long-term follow-up demonstrates that improvements in pain, pressure sensitivity, edema, bruising, and mobility can persist for over a decade after liposuction. However, gains are typically incomplete, weight change is variable, and most patients must continue conservative therapy.^32^

#### Case presentation

A 61-year-old female with a history of laparoscopic Roux-en-Y gastric bypass five years prior resulting in a subsequent weight loss of 67 kg (148 lb) was referred to plastic surgery for very large amounts of skin and adipose tissues in the lower truncal area, thighs, and legs despite bariatric surgery (Fig. 1A, B). Her body mass index (BMI) was 35.9 kg/m^2^. The patient had additional history of hypertension and bilateral knee replacements. The patient’s main concerns were the large amount of excess tissue and skin draping from the medial thighs, which caused constant rubbing and skin irritation.

**Fig. 1:**
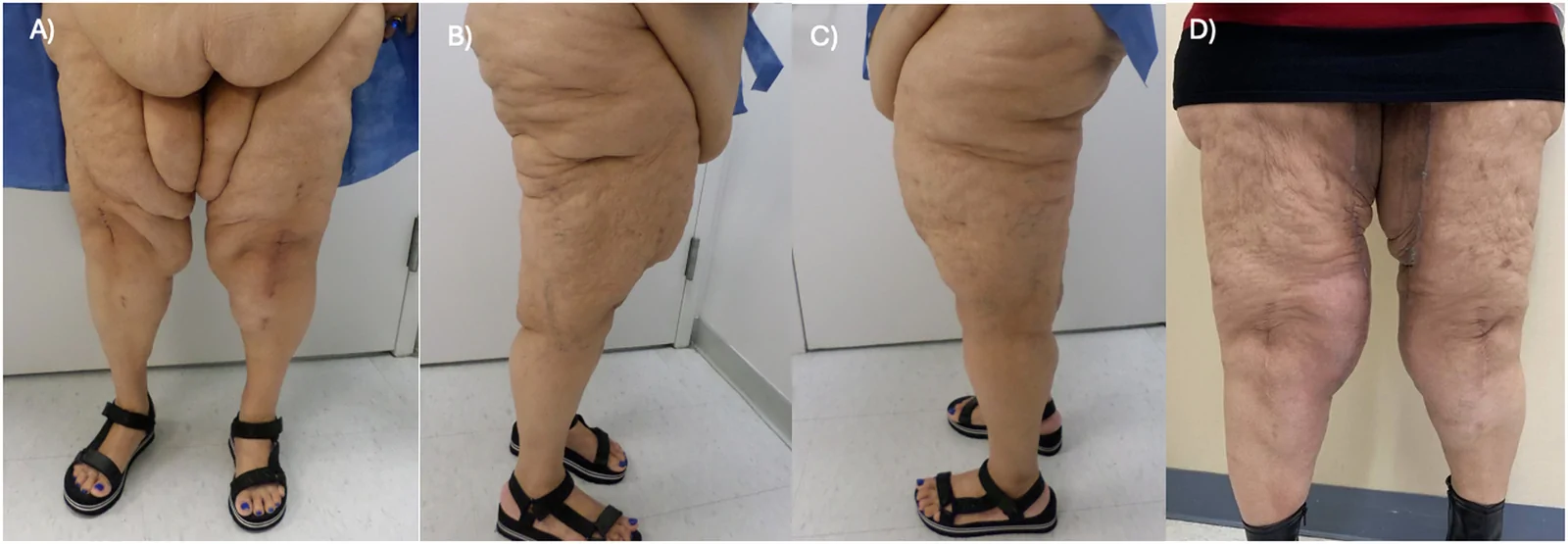
Lipedema. A-C) 61-year-old female with lipedema exhibiting symmetric enlargement of the lower trunk and extremities, including the buttocks, hips, thighs, and legs, sparing the feet. D) 6-month postoperative follow up after staged bilateral medial thighplasty following thigh liposuction.

She had trialed conservative measures, including manual lymphatic drainage, compression, and skin care, without success. To address her concerns, a staged approach was chosen beginning with tumescent-assisted liposuction in which 3 L of lipoaspirate was removed from the two thighs. Three months following this procedure, the patient elected to proceed with a panniculectomy for excess skin and tissues of the abdomen, in which 11.6 lb of tissue was removed. At three months following the initial liposuction, she continued to have large amount of excess skin (Fig. 1C) and therefore proceeded to undergo repeat liposuction and bilateral medial thighplasty. In this procedure, an additional 3 L of lipoaspirate was removed, 1.5 L per thigh, and then intraoperatively a pinch test was performed on the medial thighs to determine the extent of skin excision and the location that would allow for the final incision to settle on the medial axis of the leg. The marked skin was excised, and the incisions were closed in layers (Fig. 1D).

Our patient’s course was complicated by bilateral thigh seroma that failed to resolve with conservative management, requiring operative excision for resolution. She demonstrated improved thigh contours and reduction in medial thigh tissue excess at 6 months postoperatively.

## Non-lipedema

### Madelung disease

#### Clinical presentation and diagnosis

Madelung disease (MD), or multiple symmetric lipomatosis, is a lipid metabolism disorder characterized by the symmetric growth of non-encapsulated fatty tissue. MD primarily occurs in middle-aged men, with one systematic review finding that 95% of all patients were male with a median age of 51.4 years.^33^ Patients with MD present with diffuse adipose masses that are most concentrated in the neck, thorax, trunk, and upper extremities. In a study of 54 patients in China, Li et al. described the neck as the most affected region (100%), followed by the back (19%), shoulders (13%), pectoral region (11%), and arms (11%).^34^ MD can be categorized into three primary types depending on the affected anatomical regions – type I involves symmetric fat deposition in the neck and shoulders (described as the Madelung collar), type II involves deposition in the shoulders, arms, and chest creating a “pseudo-athletic” appearance, and type III involves deposition in the thighs and medial knees in a gynecoid distribution.^35^

Although most clinical presentations are asymptomatic, adipose masses may cause compressive symptoms, such as dysphagia and dyspnea, as well as limited mobility and range of motion. Diagnosis is clinical and is established by the characteristic clinical pattern noted above. CT and MRI, while not diagnostic, are useful for defining distribution and depth of disease, identifying structural compression, and guiding operative planning. Ultrasound provides less comprehensive preoperative information when used alone.^36^^,^^37^ Histopathologic evaluation is generally reserved for atypical lesions or cases in which malignancy cannot be excluded.^36^

#### Pathophysiology

MD is strongly associated with alcohol use and systemic comorbidities, including chronic obstructive pulmonary disease, polyneuropathy, nephropathy, liver dysfunction, diabetes, and dyslipidemia.^33^ In a study of 59 patients with MD, 85% reported a prior history of alcohol use disorder.^38^

The pathophysiology of MD remains incompletely defined and is likely heterogeneous, with hypotheses involving mitochondrial dysfunction, disruption of adrenergic lipolysis, alcohol-induced inflammation, and lymphatic vessel leakage due to hepatic dysfunction.^39^ Studies have shown impaired noradrenergic induction of genes involved in brown adipocyte proliferation and differentiation and identified brown or beige fat markers within affected tissue.^40^, ^41^, ^42^ Although mitochondrial DNA variants have been identified in selected patients, mitochondrial abnormalities are not consistently present, and conflicting respiratory-chain findings indicate that mitochondrial dysfunction is unlikely to provide a universal explanation.^41^^,^^43^ Chronic alcohol exposure may exacerbate impaired adrenergic signaling and mitochondrial metabolism but is insufficient as a primary cause as MD also occurs in patients without substantial alcohol exposure.^36^^,^^44^

#### Management

Surgery via lipectomy and/or liposuction is the primary treatment for symptomatic disease. In a systematic review of 52 articles and 106 cases, 92% of all patients were treated surgically, with 16 (15%) patients receiving both liposuction and lipectomy and only two (2%) patients receiving liposuction only.^36^ In patients who received lipectomy and liposuction combined, lipectomy alone, and liposuction alone, the rates of recurrence were 13%, 7%, and 50%, respectively.^36^ Pinto’s study found an overall recurrence rate of 39%, with time to recurrence ranging from 1.5 to 10 years depending on anatomic region.^37^ The overall rate of postoperative complications following lipectomy ranges from 7–18% and includes hematoma, seroma, local infection, skin necrosis, and facial nerve palsies.^38^^,^^45^^,^^46^

For patients without compressive or functional symptoms, observation is appropriate. Alcohol cessation is recommended, although abstinence does not reliably reverse established adipose deposits.^36^^,^^44^ Complete abstinence from alcohol, on the other hand, has been reported to reduce the rate of recurrence following surgery.^47^ Weight loss and dietary modification may improve concomitant obesity and metabolic health but generally do not reduce adipose masses.^36^ Pharmacologic approaches, including β-adrenergic agonists and fibrates, have produced apparent benefit in isolated case reports but lack compelling evidence.^48^^,^^49^ Intralipotherapy with phosphatidylcholine or deoxycholate has also been explored, although evidence is also limited and reported volume reduction has been modest.^50^^,^^51^

#### Case presentation

A 34-year-old female was referred to plastic surgery for excess symmetric fatty tissue in the upper back after a negative endocrinology workup. She noted a large excess of tissue accumulation in neck, shoulders, and back since childhood, experiencing skin irritation and issues with clothing. She reported no medical or surgery history and denied steroid or significant alcohol use. Exam was notable for large amounts of nonencapsulated fatty tissues in the bilateral upper back, shoulders, and neck (Fig. 2A, B). To address these concerns, a combined approach was chosen starting with liposuction debulking that removed 1.5 L of lipoaspirate. Three months later, this was followed by an extended bra-line back lift that involved extensive elevation of soft tissue and direct lipectomy of the upper back, posterior shoulder, and neck (total amount of tissue removed was 3.45 kg) (Fig. 2C). The patient healed uneventfully and reported greatly improved symptoms at her most recent follow up at 6 months postoperatively (Fig. 2D).

**Fig. 2:**
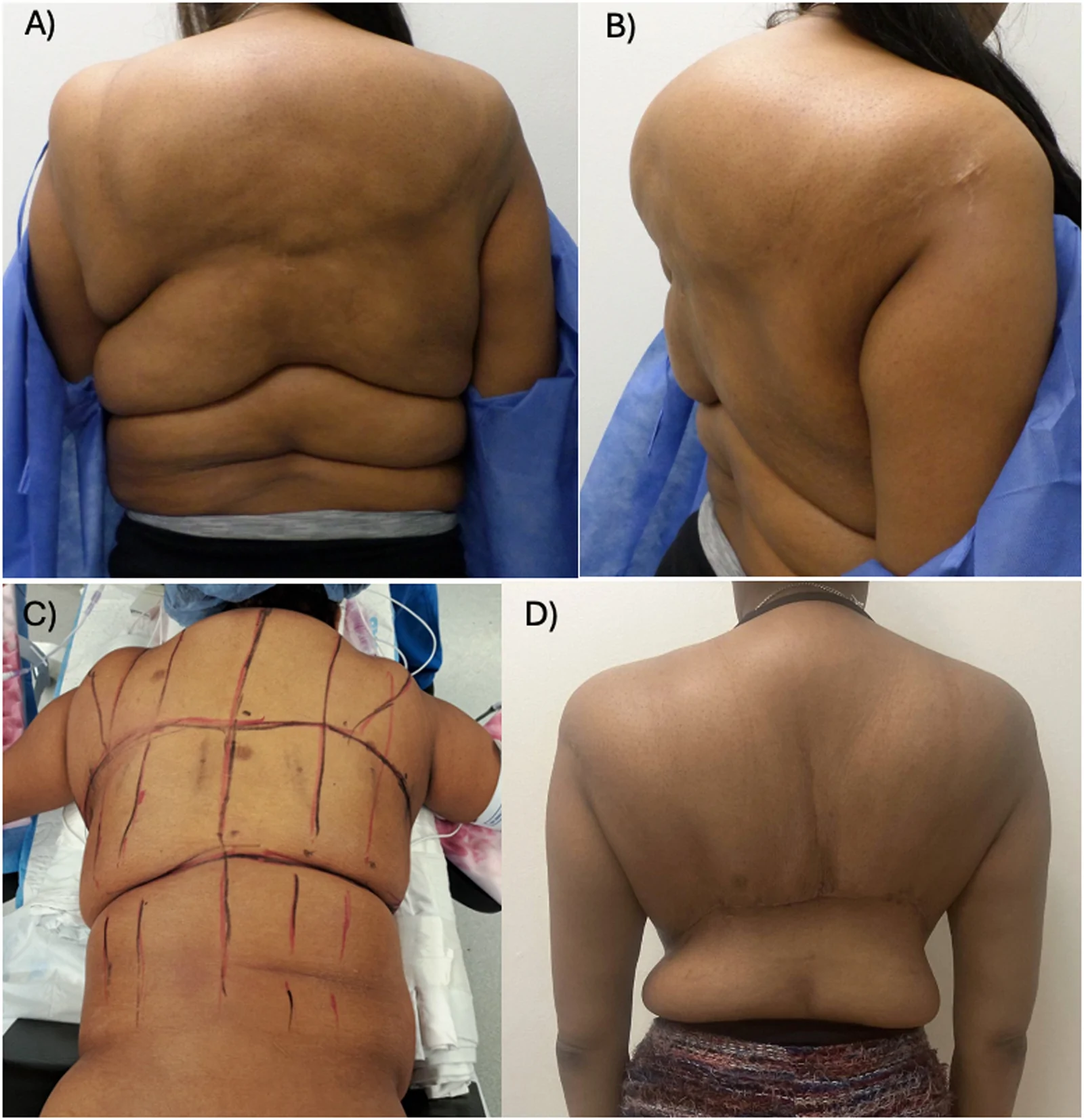
Madelung disease. A-B) 34-year-old woman with Madelung disease presenting with fatty tissue accumulation in the neck, shoulders, and back. C) Following liposuction, an extended bra-line back lift was performed to address the excess tissue in the upper back and shoulders. The two transverse markings indicate proposed skin and soft tissue excision. D) 6-month postoperative follow up.

### Familial multiple lipomatosis

#### Clinical presentation and diagnosis

Familial multiple lipomatosis (FML), or hereditary multiple lipomatosis, is a rare genetic disorder characterized by numerous benign lipomas predominantly affecting the trunk and extremities.^51^ Unlike MD—the other major form of familial lipomatosis—FML typically spares the neck and shoulder region.^52^ The lipomas in FML are generally soft, mobile, and encapsulated by a thin fibrous capsule within the subcutaneous tissue.^53^ They are usually painless but may cause local tenderness or discomfort, cosmetic deformity, or functional limitation when large or clustered near joints or neurovascular structures. ^54–56^ The disease has a typical onset in mid-adulthood and progresses slowly over several decades.^54^^,^^52^ No standardized diagnostic criteria exist for FML. Diagnosis is established primarily on clinical findings, including multiple discrete lipomas in a characteristic distribution supported by a compatible family history involving two or more generations. Genetic testing can confirm the diagnosis, and excisional biopsy may be indicated if uncertainty remains.^53^, ^55^, ^56^, ^57^ Imaging modalities such as ultrasound, MRI, or CT help define lesion depth and exclude liposarcoma in atypical presentations.^54^

#### Pathophysiology

FML is most often inherited in an autosomal dominant pattern and is frequently associated with mutations in the HMGA2 gene, which encodes a chromatin-associated protein regulating adipocyte differentiation and proliferation, although polygenic inheritance has also been proposed. ^54,56–58^ Metabolic abnormalities have not been conclusively linked to disease pathogenesis, though some studies suggest associations with altered insulin sensitivity and fluctuations in lipoma size related to weight changes.^54^^,^^56^

#### Management

Given the benign nature of the lipomas, management of FML is directed toward symptomatic relief and cosmetic improvement. Observation is appropriate for asymptomatic lipomas, while symptomatic lesions may be managed conservatively with analgesics. No medical therapy has been shown to prevent new lipoma formation or halt disease progression.^58^ An isolated case study suggested that statins may reduce lipoma size, but evidence supporting pharmacological treatment remains limited to low-quality observational data and case reports.^54^^,^^59^^,^^60^

Surgical excision remains the standard therapy, providing definitive removal but potentially resulting in extensive scarring when numerous lesions are present.^55^ Minimally invasive approaches such as limited-incision extraction, suction-assisted lipectomy, and laser-assisted lipolysis have been described as alternatives offering improved cosmetic outcomes and shorter recovery times, although supporting evidence is limited to small case series.^59^^,^^61^, ^62^, ^63^ Complete capsule removal remains critical to minimize recurrence. In a series of 30 patients, Raemdonck et al. observed lower recurrence rates at one year following excision compared to liposuction alone.^64^ Al-Basti et al. reported that treating moderate to large lipomas with liposuction-assisted extraction of the capsule through the same incision prevented recurrence over six years while yielding favorable cosmetic results.^61^ While local recurrence after complete excision appears uncommon, patients remain susceptible to developing additional lipomas over time because surgery does not address the underlying genetic predisposition, underscoring the need for long-term surveillance and higher-quality longitudinal studies. Adjunctive methods such as injection lipolysis (*e.g.*, collagenase and deoxycholate or phosphatidylcholine) and laser therapy remain under investigation but have not demonstrated sufficient evidence to replace surgical removal as the standard of care.^54^^,^^65^

#### Case presentation

A 60-year-old woman with a history of hypertension, prediabetes, and asthma presented with multiple lipomas throughout the body, including chest, abdomen, and arms. She reported a family history of multiple lipomatous masses in her mother and grandmother. The masses had been present since adolescence. She reported prior removal of lipomas in the extremities as a teenager. Exam revealed multiple lipomas in an asymmetric distribution pattern, localized to the extremities, chest, abdomen and back (Fig. 3A-C). MRI of the areas of concern demonstrated lipomatous areas in the subcutaneous tissue without radiographic evidence of liposarcoma.

**Fig. 3:**
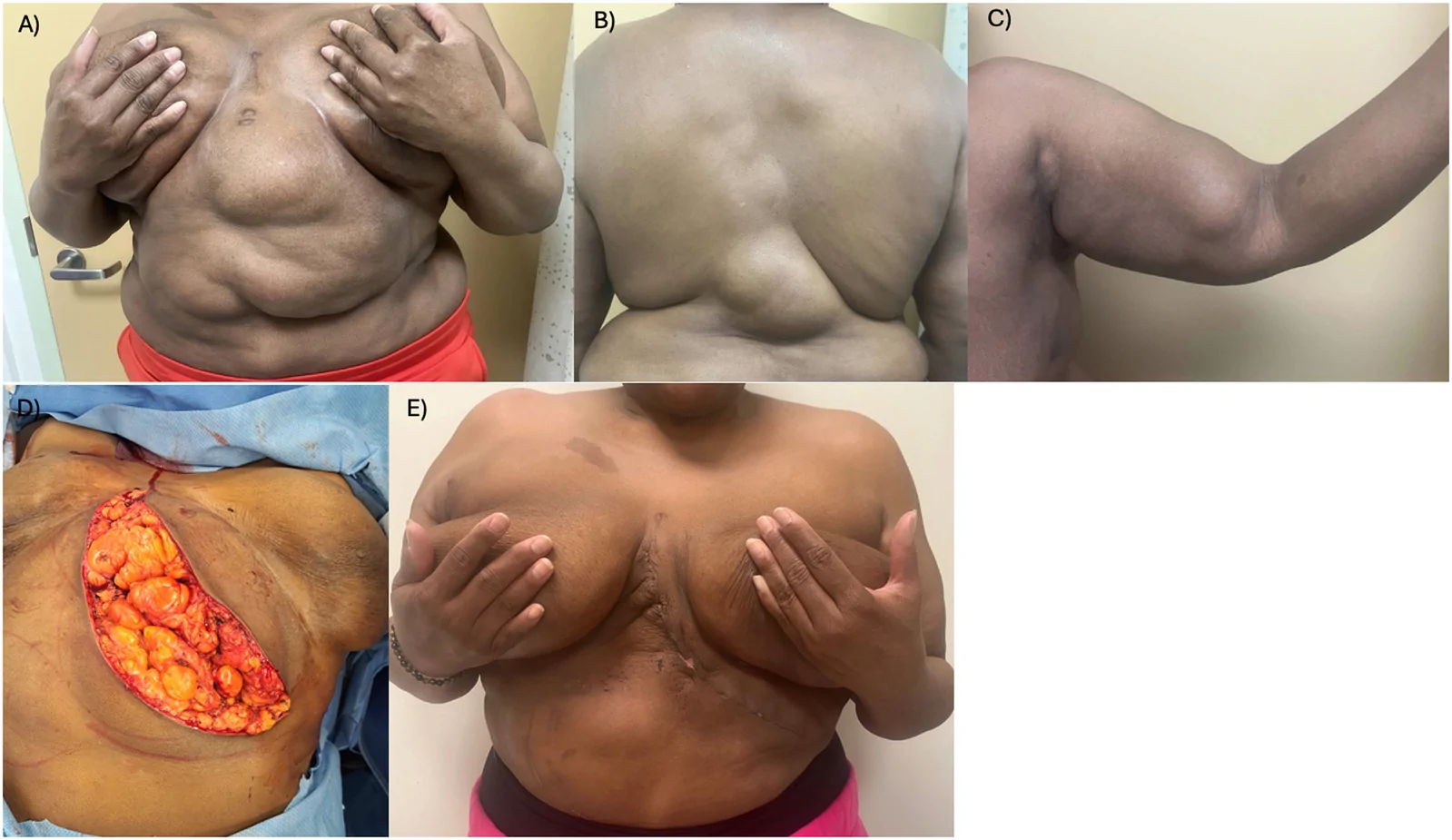
Familial multiple lipomatosis. A-C) 60-year-old female with a strong family history of lipomas presenting with several large lipomatous masses in the chest, abdomen, and arms. D) After skin flaps were raised, a large multi-lobulated lipomatous mass totaling 30×15 cm in size was revealed. E) 3-month postoperative follow up demonstrating improved contour.

Given the size, the patient desired removal of the large lipoma in her lower chest and abdomen. To address this area, an oblique incision was made, followed by skin flap elevation to fully expose the mass (Fig. 3D). A large multi-lobulated lipomatous tissue, totaling 30×15 cm in size, was removed. The skin flaps were re-draped, excess skin removed, and closure performed in a layered fashion. Pathology results were notable for mature adipose tissue. At her 3-month postoperative follow up, the patient healed uneventfully with improved contour and symptoms (Fig. 3E).

### Dercum’s disease

#### Clinical presentation and diagnosis

Dercum's disease, or adiposis dolorosa, is a rare disorder characterized by painful lipomas, primarily affecting women aged 35 to 50. The adipose masses are largely found in the trunk and extremities.^66^ Similar to lipedema, these adipose deposits are painful and can cause significant disability. While the epidemiology of the disease is largely unknown, Dercum’s disease is often found in women of middle age and is comorbid with obesity, as in lipedema.^67^ In a review of 45 patients, Asdourian et al. showed that 78% of patients were female and 69% were overweight or obese.^67^ In addition to obesity, psychiatric disorders are a common comorbidity of Dercum’s disease, with over half of the patients carrying a diagnosis of mood disorder.^67^ Dercum’s disease can be classified into four types based on location and involvement of the adipose deposits: 1) generalized diffuse form, 2) generalized nodular form, 3) localized nodular form, and 4) juxta-articular form.^68^

Dercum’s disease is a clinical diagnosis. In 2012, Hansson et al. proposed a diagnostic criterion “to be generalised overweight or obesity in combination with painful adipose tissue” in the setting of chronic, symmetrical pain.^68^ However, these criteria have not been prospectively validated and do not encompass patients with localized nodular disease or without generalized obesity. Ultrasound or MRI may characterize painful adipose nodules and exclude alternative pathology but cannot independently establish the diagnosis.^69^^,^^70^

#### Pathophysiology

Proposed pathophysiologic mechanisms include adipose inflammation and fibrosis, peripheral nerve compression or sensitization, altered adipose metabolism, and lymphatic dysfunction. However, histologic and imaging studies have not consistently identified abnormalities that distinguish painful Dercum’s tissue from obesity-associated or nonpainful adipose tissue.^69^^,^^71^, ^72^, ^73^, ^74^ A small lymphatic-imaging study demonstrated dilated vessels with sluggish flow, suggesting a possible lymphovascular contribution.^75^

#### Management

Management is primarily directed toward chronic pain control, functional improvement, and psychological support. Care is best coordinated through a multidisciplinary team that may include primary care, pain specialists, physical or lymphatic therapists, and mental-health professionals. Conventional analgesics and nonsteroidal anti-inflammatory drugs often provide incomplete relief, whereas topical, local, or intravenous lidocaine may produce temporary improvement. Isolated reports have described benefit with oral pregabalin and manual lymphatic drainage.^76^^,^^77^ Other treatments like corticosteroids, infliximab, methotrexate, and deoxycholic acid have been reported, although their efficacy has largely been restricted to case reports and series.^66^ Compression or pneumatic therapy may alleviate edema or lymphatic symptoms, although pain and hyperalgesia may limit tolerance and supporting evidence remains limited.

Liposuction is the primary surgical treatment, although surgical excision has also been shown to be a viable option for select patients.^78^^,^^79^ In a nonrandomized study of 111 patients with Dercum’s disease, including 53 who underwent liposuction, surgery was associated with lower pain levels and improved quality of life at five years, although pain relief diminished over time and causality could not be established.^72^^,^^80^

#### Case presentation

A 38-year-old female was referred for multiple painful lipomas of the upper extremity that she found bothersome and desired surgical removal. She had a history of similar lipomas in the lower extremity that were removed at an outside institution. She reported pain requiring naproxen, numbness/tingling in the arm and hand in a non-specific distribution, and anxiety/depression stemming from this issue. She had no additional medical or surgical history. History and exam were compatible with localized nodular form of Dercum’s disease with multiple lipomatous masses in the forearm that were tender and mobile (Fig. 4A-B). She underwent excision of eight distinct lipomas in the forearm and was last seen at her 1-year postoperative follow up, reporting significantly improved symptoms.

**Fig. 4:**
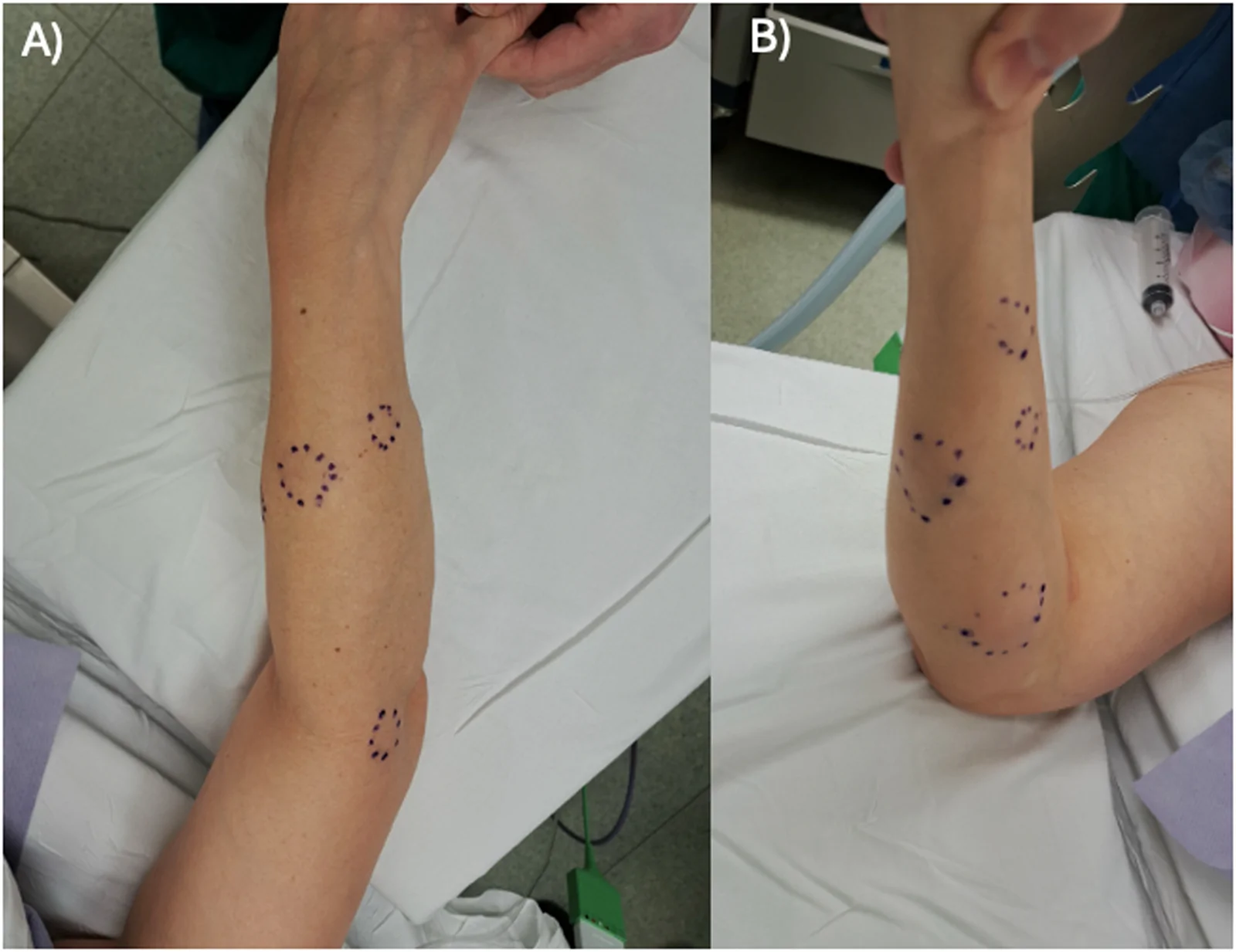
Dercum’s Disease. A-B) 38-year-old woman with a history of anxiety/depression presenting with localized nodular form of Dercum’s who subsequently underwent excision of multiple lipomas in the forearm.

## Discussion

Rare adipose disorders describe a heterogenous group of diseases that are characterized by abnormal subcutaneous adipose tissue. While they share the commonality of being resistant to traditional weight loss methods like caloric limitation and exercise, they are otherwise quite varied in epidemiology and clinical presentation. With this review, we hope to bring awareness to these underrecognized disease entities that are often misdiagnosed as obesity but are refractory to traditional weight loss measures. We provide a comprehensive review of the current literature in the pathophysiology, clinical presentation, and treatment of RADs. By presenting a patient case illustrative of each disorder and highlighting our surgical approach, we also aim to present this review as a resource for the surgical treatment of patients with RADs.

Lipedema, the most common form of RAD, predominantly affects overweight or obese women and presents as painful bilateral symmetric enlargement of the lower body, including buttocks, hips, thighs, and legs, sparing the feet and hands.^9^^,^^15^ Non-lipedema disorders include Madelung disease, familial multiple lipomatosis, and Dercum’s disease. MD is primarily found in middle-aged men with a history of alcohol use.^33^ MD presents as large, non-encapsulated fat pads in the neck, thorax, trunk, and upper extremities.^34^ FML is characterized by numerous lipomas that, in contrast to MD, are encapsulated by a thin fibrous capsule. These painless lipomas affect the trunk and extremities, sparing the neck and shoulder region.^52^^,^^53^ Dercum's primarily affects middle-aged overweight or obese women and presents as painful lipomatous masses in the trunk and extremities.^66^^,^^67^

Because of the diverse presentation, RADs are underrecognized as a disease entity and therefore undertreated. Even after an accurate diagnosis, the varied clinical presentation also presents a unique surgical challenge for plastic surgeons, as there is no one-size-fits-all approach that would successfully address all forms of RADs. From our experience treating these patients, a few guiding principles can be highlighted. Direct surgical resection should be performed in patients presenting with distinct lipomatous masses such as in Dercum’s disease and FML. Direct excision also provides the exposure necessary to remove all the capsule material to prevent recurrence in FML. In lipedema and MD, where patients present with generalized fatty deposits or non-encapsulated fat pads, liposuction is generally more effective in removing the diseased adiposity. If needed, direct lipectomy and skin excision can then follow liposuction in a staged manner to address the resulting excess skin and soft tissue. It should be recognized that liposuction carries a small potential risk for mortality, though no lipedema-specific mortality associated with liposuction has been reported.^30^

Given the rarity of these disorders, this review is comprised of studies of all levels of evidence, including case reports and series. While this may skew our findings, it is also necessary to provide a comprehensive and updated review, which include often competing hypotheses of disease pathophysiology and evolving treatment approaches. Further, while we aim to present illustrative cases highlighting our surgical experience managing patients with RADs, given disease rarity, they may not always be representative of the typical presentation. MD, for example, is strongly associated with alcohol use disorder; however, our patient has no reported history of alcohol use and represents an atypical case of MD. Finally, we are unable to comment on long-term outcomes following surgical treatment given the relatively short follow-up of 3 months to 1 year in our patient series. This limitation should especially be noted for conditions like MD, where recurrence has been reported for up to ten years postoperatively.^81^ Efforts are ongoing to continue to follow these patients to monitor long-term outcomes, including recurrence, resolution of chronic pain, and patient satisfaction.

## Conclusion

Rare adipose disorders represent a heterogenous group of diseases that are important to recognize with many having a possibility for successful surgical treatment. In this review, we discuss the pathophysiology, clinical presentation, and management of lipedema as well as rarer diseases, including Madelung disease, familial multiple lipomatosis, and Dercum’s disease. We also present a corresponding case series highlighting our surgical approach to RADs, though long-term follow up is warranted.

## Ethical approval

IRB approval was not required.

## Funding

There was no funding associated with the preparation of this manuscript.

## Declaration of competing interest

The authors disclose no relevant financial or nonfinancial interests.
